# The Histone Variant H3.3 Is Required for Plant Growth and Fertility in *Arabidopsis*

**DOI:** 10.3390/ijms25052549

**Published:** 2024-02-22

**Authors:** Xiaogang Long, Wandong Yang, Yanfang Lv, Xiaoming Zhong, Lin Chen, Qingzhu Li, Zhaopeng Lv, Yanzhuo Li, Yajun Cai, Hongchun Yang

**Affiliations:** 1 State Key Laboratory of Hybrid Rice, College of Life Sciences, Wuhan University, Wuhan 430072, China; xiaogang.long@whu.edu.cn (X.L.); ywddongji@163.com (W.Y.); lvyanfang@whu.edu.cn (Y.L.); xiaoming.zhong@whu.edu.cn (X.Z.); lin.chen@whu.edu.cn (L.C.); liqingzhu@whu.edu.cn (Q.L.); lvzhaopenglv@126.com (Z.L.); lyz096@whu.edu.cn (Y.L.); yajuncai@whu.edu.cn (Y.C.); 2 Hubei Hongshan Laboratory, Wuhan 430070, China; 3 RNA Institute, Wuhan University, Wuhan 430072, China

**Keywords:** *Arabidopsis*, histone variant H3.3, rosette growth, filament elongation, *JAZ*

## Abstract

Histones are the core components of the eukaryote chromosome, and have been implicated in transcriptional gene regulation. There are three major isoforms of histone H3 in *Arabidopsis*. Studies have shown that the H3.3 variant is pivotal in modulating nucleosome structure and gene transcription. However, the function of H3.3 during development remains to be further investigated in plants. In this study, we disrupted all three *H3.3* genes in *Arabidopsis*. Two triple mutants, *h3.3cr-4* and *h3.3cr-5,* were created by the CRISPR/Cas9 system. The mutant plants displayed smaller rosettes and decreased fertility. The stunted growth of *h3.3cr-4* may result from reduced expression of cell cycle regulators. The shorter stamen filaments, but not the fertile ability of the gametophytes, resulted in reduced fertility of *h3.3cr-4*. The transcriptome analysis suggested that the reduced filament elongation of *h3.3cr-4* was probably caused by the ectopic expression of several *JASMONATE-ZIM DOMAIN* (*JAZ*) genes, which are the key repressors of the signaling pathway of the phytohormone jasmonic acid (JA). These observations suggest that the histone variant H3.3 promotes plant growth, including rosette growth and filament elongation.

## 1. Introduction

The eukaryotic genomic DNA is tightly wrapped around histones to form nucleosomes as the fundamental unit of chromatin [1]. Three major variants evolved from the histone H3 family in *Arabidopsis*: the canonical histone H3.1, the replacement histone H3.3, and the centromeric histone CenH3/CENP-A [2,3]. Histones are highly conserved. Only four residue variations exist between H3.3 and H3.1 in *Arabidopsis* [4]. Canonical histone H3.1 is transcribed during the S-phase of the cell cycle and assembled into nucleosomes behind the replication fork to package newly synthesized DNA [5,6]. By contrast, the histone variant H3.3 is expressed throughout the cell cycle and can be incorporated into chromatin independently of DNA synthesis. Incorporating histone variants can profoundly change nucleosome properties and affect chromosome segregation, as well as DNA transcription, replication, and repair [7,8]. H3.3 is typically associated with transcriptionally active regions, whereas H3.1 is enriched in the silenced chromatin regions [9,10,11,12]. The vascular-plant-specific phenylalanine residue at the N-terminal tail and the core region of H3.1 are critical for its genomic distribution [6]. H3.3 is more specifically enriched at transcriptional end sites (TES) of genes and is associated with active transcription in *Arabidopsis*. The tight link between H3.3 replacement and transcription indicates an important function of H3.3 during gene transcription, and hence developmental transition [13,14,15].

Histone H3.3 exhibits a unique genetic pattern in which two or more genes encode identical proteins. In mice, H3.3 is required for fertility. Even knockout of one of the coding genes reduces viability and fertility [16,17,18]; complete loss of H3.3 results in developmental retardation and early embryonic lethality [19]. In *Drosophila*, the absence of H3.3 leads to partial lethality and sterility [20,21]. Quite remarkably, the complete depletion of H3.3 is still viable and fertile in *Caenorhabditis elegans* [22]. In *Arabidopsis*, the *H3.1* genes do not contain any intron, whereas there are introns in each *H3.3* gene. Three *H3.3* genes, *HISTONE 3 RELATED 4* (*HTR4*), *HTR5*, and *HTR8*, are identified in the *Arabidopsis* genome. Among them, *HTR4* and *HTR5* are tandemly located at chromosome four. There is no obvious developmental defect of double *H3.3* mutants [15]. In *htr4 htr8* double knockout background, further knockdown of *HTR5* by RNA interference (RNAi) leads to reduced growth, early flowering, and slightly reduced fertility [15,23]. Further analysis suggests that H3.3 maintains the active chromatin state at the flowering repressor locus *FLOWERING LOCUS C* (*FLC*), leading to floral repression [23]. A complete knockout of *H3.3* results in severe defects in germination, but the embryogenesis is similar to the wild type (WT) [24]. However, the role of H3.3 in the other developmental processes is very limited.

Phytohormones tightly control plant growth and development. The phytohormone jasmonic acid (JA) was initially recognized as a stress hormone that regulates plant adaptation to biotic and abiotic stresses [25]. In recent years, increasing studies have shown that the JA signaling pathway also plays essential roles in many aspects of plant development, especially in promoting stamen filament elongation [26,27,28,29,30]. Ectopic expression of JA biosynthesis genes promotes stamen filament elongation [28]. Some mutants with impaired JA biosynthesis or signaling display shorter filaments [30,31,32,33]. Other phytohormones, including auxin and gibberellin (GA), regulate JA biosynthesis to control filament elongation [34,35,36]. Thus, the JA pathway is a hub for stamen elongation [26].

JASMONATE-ZIM DOMAIN (JAZ) proteins are critical regulators of the JA signaling pathway. They inhibit the expression of downstream responsive genes [37]. Upon perceiving bioactive JAs, the JA receptor Coronatine Insensitive 1 (COI1) targets JAZs to the Skp1/Cullin/F-box SCF^COI1^ complex for ubiquitination. The ubiquitinated JAZs are degraded through the 26S proteasome pathway, releasing downstream transcription factors (TFs) to regulate JA responses [38]. For example, the degradation of JAZs releases the basic helix–loop–helix (bHLH)-MYB complex to activate the expression of downstream genes, which are essential for filament elongation [38,39,40]. Whether H3.3 regulates the JA signaling pathway is unknown. In this study, we created two *H3.3* mutants, which displayed reduced growth and shorter filaments. The shorter filaments of *h3.3* mutants led to reduced fertility. Gene expression analysis suggested that the cell cycle regulators and JA signaling pathway genes (*JAZs*) may play a role in H3.3-mediated developmental regulation in *Arabidopsis*.

## 2. Results

### 2.1. Generation of h3.3 Mutants by CRISPR/Cas9

**Figure 1:**
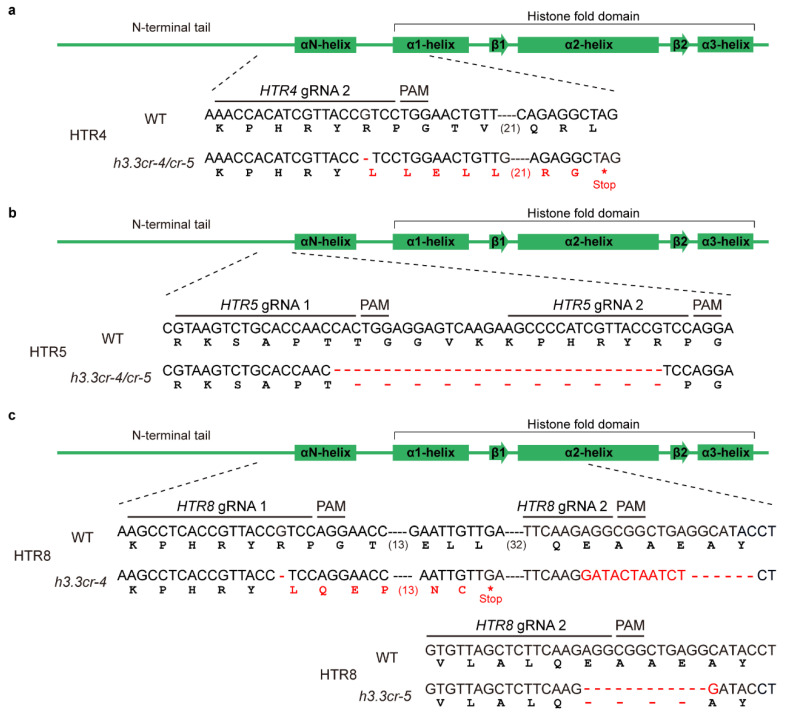
Identifying mutations in *h3.3cr-4* and *h3.3cr-5* mutants. (**a**–**c**) The mutation site of *HTR4* (**a**), *HTR5* (**b**), and *HTR8* (**c**). The green boxes and lines display the protein domain structure of H3.3. The N-terminal tail and the α-helixes of the histone fold domain are shown. The sequence marked with a black line represents the small guide RNA (gRNA) sequence and protospacer-adjacent motif (PAM) sequence. The red nucleotide or amino acid indicates the mutation site, and the red asterisk indicates the stop codon. Green display the schematic picture of the protein domain structure of H3.3. Red display the mutation nucleotide or amino acid.

In *Arabidopsis*, histone variant H3.3 is encoded by three genes, *HTR4* (AT4G40030), *HTR5* (AT4G40040), and *HTR8* (AT5G10980), with very high sequence similarity. *HTR4* and *HTR5* are tandemly distributed at chromosome 4, and HTR8 is located at chromosome 5. To elucidate the function of histone variant H3.3 in *Arabidopsis*, we designed a CRISPR/Cas9-based strategy to edit *HTR4*, *HTR5*, and *HTR8*. Two target sites for each gene were designed among the relatively similar sequences, facilitating one small guide RNA (gRNA) to simultaneously target multiple genes (Figure S1a, Table S1). We constructed a CRISPR/Cas9 vector containing six gRNAs and targeting all three *H3.3* genes (Figure S1b). Through progeny screening, we obtained two types of *h3.3* triple mutants, *h3.3cr-4* and *h3.3cr-5*. The mutations of *HTR4* and *HTR5* were the same in *h3.3cr-4* and *h3.3cr-5*. A single DNA base deletion within *HTR4* at the gRNA2 site led to a frameshift and a premature translational termination at the α1-helix region. The mutations disrupted the histone fold domain (Figure 1a). Thus, we concluded that the *HTR4* mutation in *h3.3cr-4* and *h3.3cr-5* abolished the function of HTR4. A 33-base deletion between gRNA1 and gRNA2 was identified within *HTR5*, which led to 11-amino acid deletion at the N-terminal tail of HTR5 and did not disrupt the core folding domain (Figure 1b). The expression of the mutated *HTR5* in *h3.3cr-4* and *h3.3cr-5* displayed similar expression levels as WT (Figure S1c) assayed by semi-quantitative RT-qPCR, suggesting that *HTR5* could be a weak mutation. The mutations of *HTR8* in *h3.3cr-4* and *h3.3cr-5* were different. A single base deletion at the gRNA1 within *HTR8* led to a premature translational termination in *h3.3cr-4*, which blocked the translation of the histone fold domain (Figure 1c). A 12-base deletion within *HTR8* resulted in a 4-amino-acid deletion at the histone fold domain in *h3.3cr-5* (Figure 1c).

### 2.2. h3.3 Mutants Exhibit Severe Growth Defects

**Figure 2:**
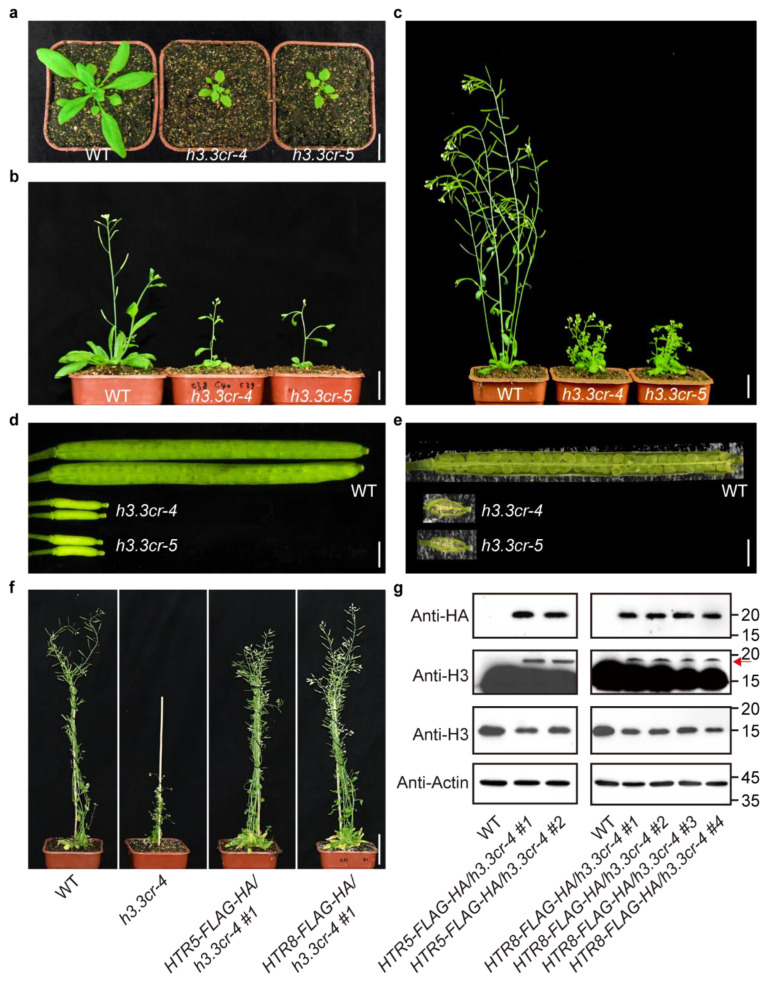
H3.3 is required to maintain rosette growth and plant fertility. (**a**–**c**) Plant morphologies of wild type (WT), *h3.3cr-4*, and *h3.3cr-5* growing under long-day (LD) conditions for 24 days (**a**), 33 days (**b**), and 52 days (**c**). Scale bar, 2.5 cm. (**d**) Siliques of WT, *h3.3cr-4*, and *h3.3cr-5*. Scale bar, 1 mm. (**e**) Opened siliques of WT, *h3.3cr-4*, and *h3.3cr-5*. Scale bar, 1 mm. (**f**) The transgenic lines complemented the *h3.3cr-4* developmental defects. WT, *h3.3cr-4*, *HTR5-FLAG-HA/h3.3cr-4*, and *HTR8-FLAG-HA/h3.3cr-4* from left to right. Scale bar, 4 cm. (**g**) The expression levels of transgenic H3.3 (red arrow indicates) with FLAG-HA fusion and endogenous H3 in the transgenic lines detected by Western blot. Actin was used as the loading control. The molecular weight (kilodalton, kd) is listed on the right of each panel.

The *h3.3cr-4* and *h3.3cr-5* plants displayed much smaller rosettes at all the developmental stages, and shorter plants at the flowering and mature stages compared with WT (Figure 2a–c). The reduced growth of *h3.3cr-4* and *h3.3cr-5* demonstrated that the proper function of H3.3 is required for plant development in *Arabidopsis*. We also found that the mutant plants had severely reduced fertility, showing very short siliques (Figure 2d). These siliques had no developing seeds (Figure 2e), suggesting perturbed fertilization in *h3.3cr-4* and *h3.3cr-5*. Because of similar developmental defects in *h3.3cr-4* and *h3.3cr-5* (Figure 2a–e), we selected *h3.3cr-4* in the following analysis. We performed complementation assays to further confirm that the observed growth defects of *h3.3cr-4* were caused by disrupted H3.3. A genomic region containing 1.2 kilobases of promoter and a full-length genomic sequence of *HTR5* was fused with tandem fluorescence activating and absorbing (FLAG) and hemagglutinin (HA) epitope tags. The *HTR5-FLAG-HA* construct was transformed into *htr4 htr5 htr8^+/−^* plants derived from *h3.3cr-4*. The homozygous *HTR5-FLAG-HA/h3.3cr-4* were selected for further analysis. The *HTR5-FLAG-HA* transgene was able to rescue all the developmental defects of *h3.3cr-4*, displaying WT-like rosette size, plant height, and silique length (Figure 2f). Similarly, *HTR8-FLAG-HA/h3.3cr-4* plants also displayed WT-like growth (Figure 2f). The complementation experiments demonstrated that the mutations of *H3.3* are responsible for the pleiotropic phenotypes observed in *h3.3* plants. Next, we measured the endogenous H3 protein level and transgenic H3.3 level in the transgenic complementation lines. The endogenous H3 level was lower than WT (Figure 2g), suggesting reduced total H3 in *h3.3cr-4*. The transgene could be detected by anti-HA and anti-H3 with a longer exposure time, supporting the restoration of the developmental defects in *HTR5-FLAG-HA/h3.3cr-4* and *HTR8-FLAG-HA/h3.3cr-4*. However, the total H3 levels in *HTR5-FLAG-HA/h3.3cr-4* and *HTR8-FLAG-HA/h3.3cr-4* were lower than in WT (Figure 2g).

### 2.3. H3.3 Promotes the Expression of the Cell Cycle Regulators

**Figure 3:**
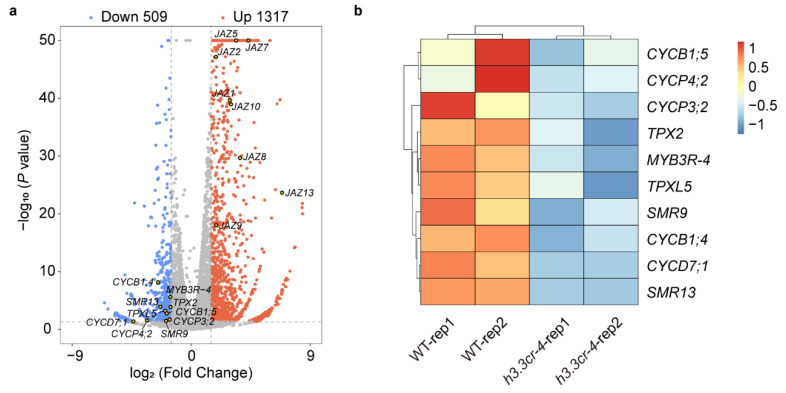
The transcriptome analysis of *h3.3-cr4*. (**a**) Volcano plot displays the DEGs between WT and *h3.3cr-4*. Blue dots show down-regulated genes, gray dots show genes without significant change, and red dots show up-regulated genes. The x-axis represents log_2_ (fold change), and the y-axis represents -log_10_ (*p*-value). (**b**) A comparison of several cell cycle regulators in the rosette leaves of WT and *h3.3cr-4*. The data are displayed as a heatmap generated with scale = row dependent on transcripts per million (TPM) values. The color scale is on the right.

To uncover the gene expression basis of the developmental defects in the *H3.3* mutants, 26-day-old rosette leaves were collected for transcriptome analysis by RNA-seq. Differentially expressed genes (DEGs) between WT and *h3.3cr-4* were identified as the multiple-test corrected *p*-value < 0.05 and absolute log_2_ (fold change) >1.5. In total, 1826 DEGs were identified, including 1317 up-regulated genes and 509 down-regulated genes (Figure 3a, Table S2). We found that the expression levels of several cell cycle regulators were decreased, such as *CYCLIN B1;4 (CYCB1;4*), *CYCB1;5*, etc., in *h3.3cr-4* (Figure 3b, Table S2), which is consistent with the reduced growth of *h3.3cr-4*. The gene expression analysis indicated that the reduced expression of cell cycle genes could be the cause of a smaller *h3.3cr-4* plant.

### 2.4. h3.3cr-4 Could Produce Normal Male and Female Gametophytes

**Figure 4:**
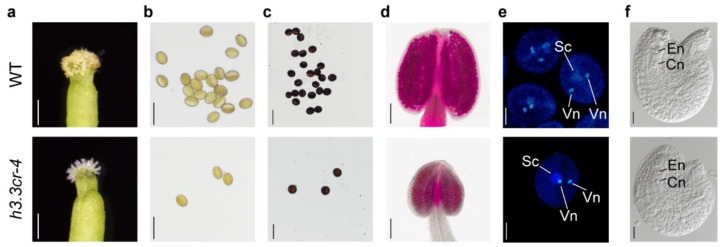
*h3.3cr-4* was able to produce normal and viable pollens and ovules. (**a**) Stigmas of WT and *h3.3cr-4* after flowering. Scale bar, 250 μm. (**b**) Pollen grains of WT and *h3.3cr-4*. Scale bar, 50 μm. (**c**) I_2_-KI stained pollens in WT and *h3.3cr-4*. Scale bar, 100 μm. (**d**) Alexander staining of the anthers in WT and *h3.3cr-4*. Scale bar, 100 μm. (**e**) DAPI staining of pollens in WT and *h3.3cr-4*. The sperm cell (Sc) and vegetative nuclei (Vn) of each pollen are labeled. Scale bar, 25 μm. (**f**) Images of ovules at FG7 stage. The central cell nuclei (Cn) and egg cell nuclei (En) are labeled in each ovule. Scale bar, 20 μm.

Then, we explored the developmental basis of the infertility phenotype in *h3.3cr-4* and *h3.3cr-5*. We found that the stigma were full of pollens after flowering in WT (Figure 4a). There was almost no pollen on the stigma of the *h3.3cr-4* plants, showing a typically unpollinated stigma (Figure 4a). This supported our previous observation that there were no developing seeds in the pistils of *h3.3cr-4* (Figure 2e). Successful fertilization relies on the proper development of male and female gametes and every step involved in double fertilization [41,42]. Next, we checked the development of male and female gametes in *h3.3cr-4*. First, although the anther of *h3.3cr-4* was smaller than that of WT, we still could see the opened anther and released pollen grains on the surface of the anther (Figure S2). Consistent with smaller anthers, fewer pollen grains were observed in *h3.3cr-4* compared to WT; however, the shape was similar to WT (Figure 4b). And these pollens could be stained using I_2_-KI solution to dark brown (Figure 4c), indicating that the starch is produced in the mature pollen grains in *h3.3cr-4*. Pollen viability was determined via Alexander staining. Almost all of the pollen grains were stained red, indicating that the pollens were viable in *h3.3cr-4* (Figure 4d). Furthermore, 4′,6-diamidino-2-phenylindole (DAPI) staining showed two bright, intensely stained sperm cells and one diffuse, weakly stained vegetative nucleus in pollens of WT and *h3.3cr-4* (Figure 4e), showing an intact meiotic division process and completed trinucleate stage. These observations displayed that WT-like pollens were produced in *h3.3cr-4*. Second, we observed the interaction of the pollen and stigma after manual pollination. Like WT, pollens from *h3.3cr-4* could change from oblate to round when artificially placed on the pistil stigma (Figure S3a), suggesting that the pollen of *h3.3cr-4* could hydrate the stigma. We performed aniline blue staining to observe the pollen germination situation. The flowers were emasculated and artificially pollinated 24 h later, followed by aniline blue staining. The pollen from *h3.3cr-4* could germinate on the stigma, and pollen tubes could normally elongate like WT (Figure S3b). Third, we analyzed the development of *h3.3cr-4* ovules. Flowers were manually emasculated. After 36 h, FG7-stage ovules were observed to contain normal-looking nuclei in the egg cells and central cells in *h3.3cr-4* (Figure 4f), showing that the *h3.3cr-4* could produce WT-like female gametophytes.

### 2.5. Hand Pollination Rescued the Fertility Defects of h3.3cr-4

**Figure 5:**
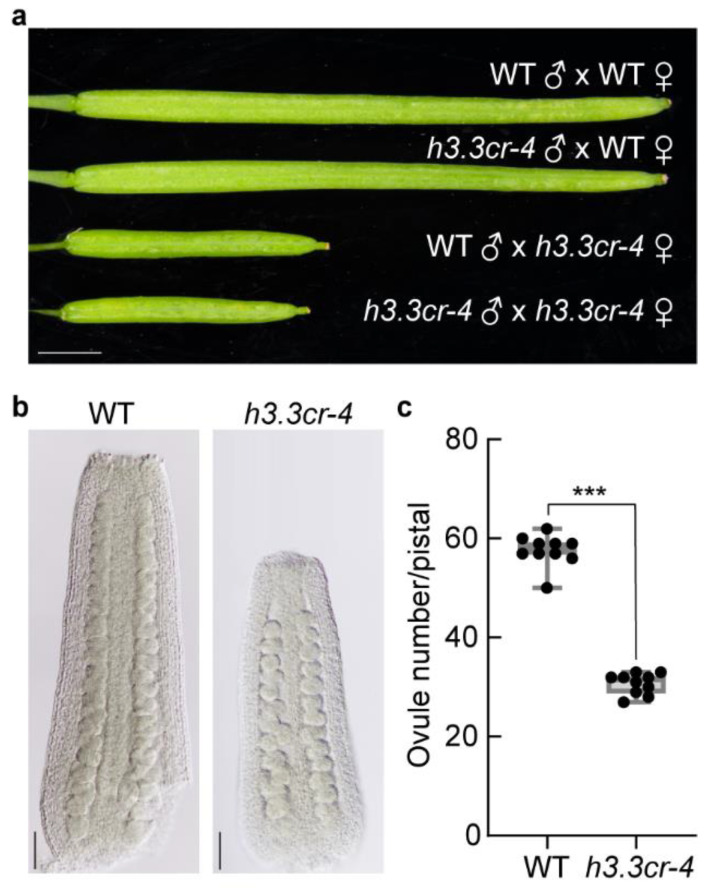
The fertile pollen and ovule of *h3.3cr-4* due to hand pollination. (**a**) Silique from reciprocal crosses between WT and *h3.3cr-4*. Scale bar, 2 mm. (**b**) Image of pistil after 36 h of emasculation in WT and *h3.3cr-4*. Scale bar, 50 μm. (**c**) Ovule number in each pistil. Significant differences were determined by unpaired two-tailed Student’s *t*-tests (*** *p* < 0.001).

To explore the cause of infertility in *h3.3cr-4*, we performed hand pollination between WT and *h3.3cr-4*. No matter whether *h3.3cr-4* supplied the stigma or pollen, elongated siliques were observed (Figure 5a), which were longer than the naturally grown *h3.3cr-4* but shorter than WT (Figure 2d). However, we could pistils full of developing seeds, indicating that the shorter siliques were caused by reduced ovule numbers in each pistil of *h3.3cr-4.* It was confirmed by differential interference contrast (DIC) observation that fewer ovules were observed in *h3.3cr-4* pistils (Figure 5b,c). Therefore, the infertility of *h3.3cr-4* is not caused by developmental defects of male and female gametophytes; a barrier between pollen and stigma could cause reduced fertility.

### 2.6. H3.3 Is Required for Stamen Filament Elongation

**Figure 6:**
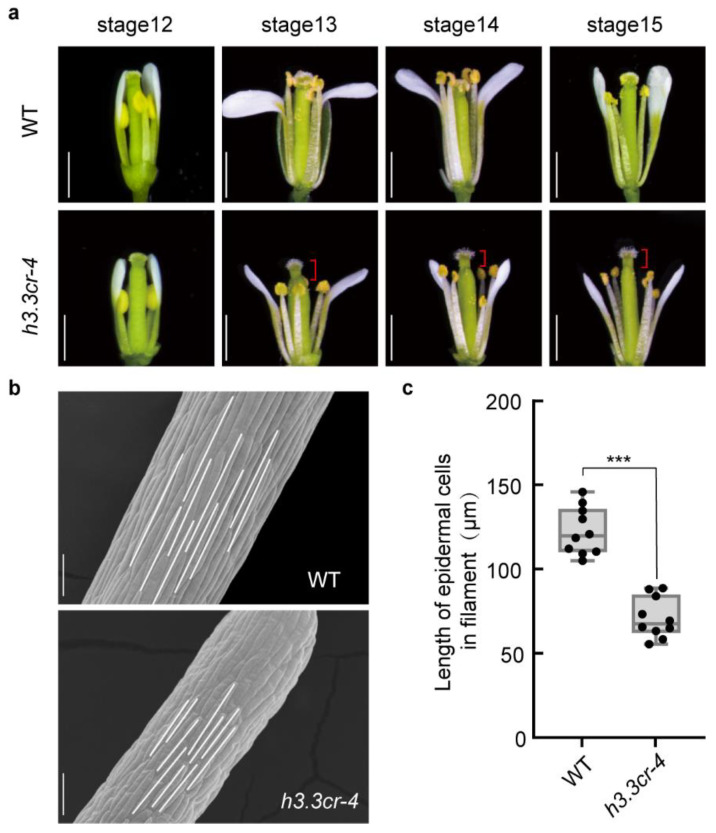
Defective stamen filament elongation in *h3.3cr-4*. (**a**) Opened flowers are shown from flower stages 12–15 of WT and *h3.3cr-4*. The red bracket indicates the gap between the stigma and the anther. Scale bar, 1 mm. (**b**) Filament epidermal cells of WT and *h3.3cr-4* under scanning electron microscope (SEM), scale bar = 50 μm. (**c**) Quantification of the length of the filament epidermal cells (**b**). Significant differences were determined by unpaired two-tailed Student’s *t*-tests (*** *p* < 0.001).

We thus carefully examined pistil development in *h3.3cr-4* across flower development stages 12–15, which cover the pollination process. Compared to WT, the filament elongation was severely perturbed in *h3.3cr-4* at each observed stage, resulting in a remarkably shorter filament than the pistil (Figure 6a). We also found that the epidermal cell of *h3.3cr-4* filament was significantly shorter than WT, as detected by scanning electron microscope (SEM) observation (Figure 6b,c). Sufficient contact of the pistils and stamens is a prerequisite for pollen dropping to the stigma [43]. The infertile siliques of *h3.3* were probably caused by the shorter filament, which reduced the accessibility of pollen to stigma and led to incomplete double fertilization.

### 2.7. H3.3 Represses the Expression of JAZ Genes

**Figure 7:**
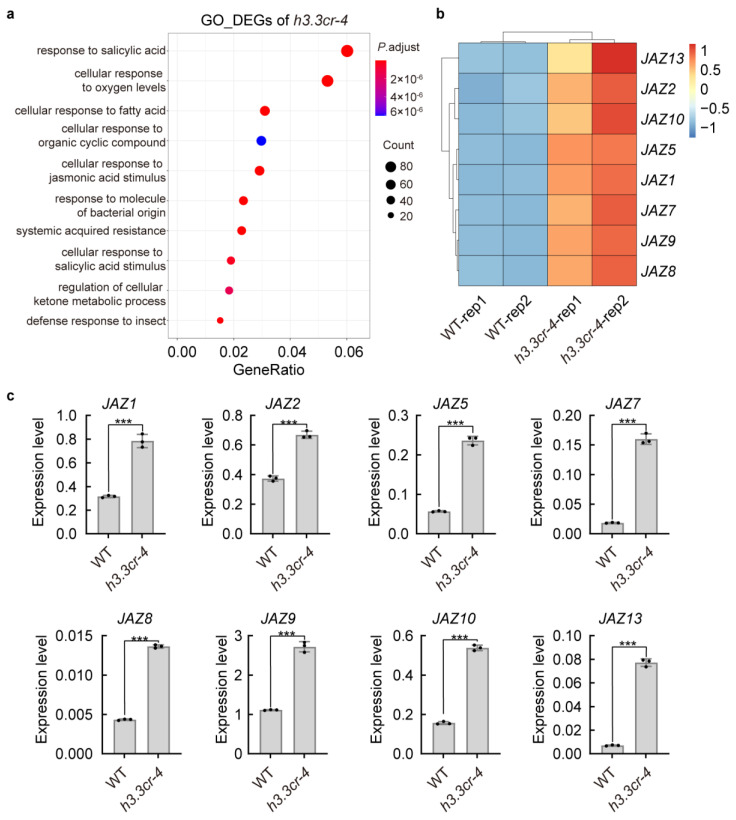
Increased expression of several *JASMONATE-ZIM DOMAIN (JAZ)* family genes in *h3.3cr-4*. (**a**) The enrichment of the top 10 significant GO terms in DEGs between WT and *h3.3cr-4*. The x-axis shows the enrichment degree of each GO term. The size and color of each point indicates the gene number in the given GO term and the *p* adjustment, respectively. (**b**) Expression comparison of *JAZ* genes in WT and *h3.3cr-4* according to RNA-seq data. The data are displayed as a heatmap generated with scale = row dependent on transcripts per million (TPM) values. The color scale is on the right. (**c**) Expression levels of *JAZ* genes in the inflorescence of WT and *h3.3cr-4* measured by RT-qPCR. Values are mean ± SD (n = 3). The significant differences were determined via unpaired two-tailed Student’s *t*-test (*** *p* < 0.001) and are represented as asterisks.

To gain possible molecular information on the impaired filament elongation of *h3.3cr-4*, we subjected the DEGs to gene ontology (GO) term enrichment analysis. The top 10 enriched GO pathways revealed a large variety of response processes, including genes involved in salicylic acid (SA) and JA signaling pathways (Figure 7a, Table S3). Consistent with the defense roles of these two phytohormones in biotic and abiotic stresses, the downstream response genes were also identified in the GO terms (Figure 7a, Table S3). Several independent studies have demonstrated that the phytohormone JA is required for stamen filament elongation [28,32,44,45,46]. To understand how stamen filament elongation is regulated in *h3.3cr-4*, we focused our analysis on the JA signaling pathway. We found that 8 of 13 *JAZ* genes were up-regulated in our transcriptome analysis (Figure 7b, Table S2). This suggests that H3.3 may regulate stamen filament elongation by changing the expression of *JAZ* genes. We confirmed the ectopic expression of these eight *JAZ* genes in the inflorescences of *h3.3cr-4* via real-time quantitative PCR (RT-qPCR). All these *JAZ* genes were also increased (Figure 7c), which is consistent with the RNA-seq data. This result indicates that the histone variant H3.3 may repress the expression of *JAZ* genes to ensure the proper development of stamen filaments.

## 3. Discussion

The histone fold domain is central to histone deposition [8]. The residues located at the N-terminal tail may coordinate with the residues in the H3.1 fold domain to ultimately determine its distribution pattern in *Arabidopsis* [6]. Research suggests that the residues located at the N-terminal tail are influenced by versatile post-translational modifications (PTMs) [8,47,48]. Previous research created an *H3.3* knockout mutant by combining a CRISPR/Cas9 double mutant *htr4 htr5* with a T-DNA insertion mutant *htr8* in *Arabidopsis*. *HTR4*, *HTR5*, and *HTR8* mutations (*h3.3ko*) all disrupted the core histone folding domain [15]. Knockout of *H3.3* causes germination and growth defects, as well as difficulty developing into the flowering stage [24]. That leaves uncertainty regarding whether and how the H3.3 regulates the process of sexual reproduction in plants. Here, we obtained two types of *h3.3* triple mutants that displayed similar phenotypes. The mutations of *HTR4* and *HTR8* were located in the core folding domain (Figure 1a,c). The mutation of *HTR5* disrupted the N-terminal tail and remained full of the core folding domain (Figure 1b), suggesting that *h3.3cr-4* and *h3.3cr-5* could be weak mutants. This is consistent with the weaker developmental defects in *h3.3cr-4* and *h3.3cr-5* compared to *h3.3ko*.

H3.1 differs from H3.3, with only four amino acids in *Arabidopsis*. It has been reported that expressing *HTR13* (an H3.1-coding gene) under the *HTR5* promoter or expressing *HTR5* using the *HTR13* promoter cannot complement the phenotype of the *h3.3ko* mutant [24]. These results suggest that histone variant H3.3 and its expression pattern are required for its unique function in many developmental processes. Western blot results showed decreased endogenous and total H3 levels in *HTR5-FLAG-HA/h3.3cr-4* and *HTR8-FLAG-HA/h3.3cr-4* complementation lines (Figure 2g). These observations together suggest that the expression pattern, but not the absolute level, is crucial for the function of H3.3 in *Arabidopsis*. This feature may have species specificity. In mice, *Caenorhabditis elegans,* and *Arabidopsis*, complete loss of H3.3 results in distinct phenotypes [16,17,18,22]. This suggests a diverged function of H3.3 in different species.

*h3.3* plants displayed pleiotropic developmental defects during different development stages. The mutant plants showed smaller rosettes (Figure 2a), shorter plants (Figure 2b), smaller anthers (Figure 4d), fewer pollen grains (Figure S2), and fewer ovules (Figure 5b and c) compared to WT. These phenotypes reflect the overall growth retardation of the plant. We found that a few cell cycle genes were down-regulated in *h3.3cr-4*, including *CYCB1;4* and *CYCB1;5* (Figure 3b). Natural variations in *CYCB1;4* have been shown to influence seed size through regulating the cell cycle in *Arabidopsis* [49]. This supports our hypothesis that inhibiting the expression of several cell cycle regulators results in stunted growth in *h3.3* mutants.

The JA signaling could promote stamen filament epidermal cell elongation to promote filament growth in *Arabidopsis* [26,28,50]. Other hormonal pathways or proteins, such as GA [34], auxins [35], and the homeotic protein AGAMOUS [51], directly or indirectly regulate JA signaling to control filament elongation. JAZ repressor proteins are central to the JA signaling cascades [38]. JA biosynthesis triggers JAZ degradation to control the expression levels of transcription factors *MYB21* and *MYB24* and, thereby, filament elongation [39]. The plants with reduced JA levels, such as JA biosynthesis-deficient mutants or those overexpressing the JA catabolism gene *CYP94B3*, result in shorter filaments [30,44,52]. Mutation of the JA receptor *COI1* also results in reduced growth of the filaments [53]. Overexpression of a non-functional *JAZ10* splice variant (*JAZ10.4*) also disrupts filament elongation [54]. All of these observations support the essential role of JA signaling in filament elongation. Here, we found that the expression levels of eight *JAZ* genes were increased in the inflorescence of *h3.3cr-4* (Figure 7c). Consistent with the ectopically expressed *JAZs*, the *h3.3cr-4* plants showed reduced filament growth and infertility (Figure 2a–e). Therefore, our data supply new evidence that H3.3 represses the expression of *JAZs* to promote filament elongation, thereby ensuring plant fertility. During flower development, Polycomb repressive complex 2 (PRC2) mediates repressive chromatin modifications histone H3 lysine 27 trimethylation (H3K27me3) to silence the expression of *JAZ1* [37]. In addition, H3.3 is required in order to properly establish H3K27me3 at the promoters of developmentally regulated genes [47,55]. H3.3 may inhibit the expression of *JAZ* genes by repression-associated H3K27me3.

## 4. Materials and Methods

### 4.1. Plant Materials and Growth Conditions

All wild-type (WT), mutant, and transgenic plants were in a Columbia-0 (Col-0) background. The seeds were surface sterilized, sown on standard half-strength Murashige and Skoog (MS) with 1% sucrose, and kept at 4 °C in the dark for 3 days. Seedlings were planted and grown in long photoperiod conditions (16 h light, 8 h dark at a constant temperature of 22 °C).

### 4.2. Plasmid Constructs

The *H3.3* CRISPR/Cas9 genome editing vector was constructed as previously described [56]. The coding sequences (CDS) of three *H3.3* genes were obtained from the *Arabidopsis* genome (TAIR10) and aligned. Two target sequences were designed for each gene. Primers were obtained on the website (http://skl.scau.edu.cn, accessed on 11 October 2023) [57]. After two rounds of PCR reaction, expression cassettes were constructed and then assembled by means of Golden Gate ligation. The *H3.3* CRISPR/Cas9 genome editing vector was transformed into WT. To create *HTR5-FLAG-HA* and *HTR8-FLAG-HA* constructs, the full-length genomic fragment of *HTR5* or *HTR8* without a terminator sequence was amplified from WT genomic DNA. The *FLAG-HA* tag was inserted before the translational stop codon, and the T3A terminator sequence was inserted after the translational stop codon. Then, these two vectors were transformed into *htr4 htr5 htr8^+/−^* plants derived from *h3.3cr-4*. All the primers are listed in Table S1.

### 4.3. Western Blot

To detect the expression levels of H3.3 protein, 11-day-old seedlings of WT, *HTR5-FLAG-HA/h3.3cr-4*, and *HTR8-FLAG-HA/h3.3cr-4* were harvested and ground to a fine powder in liquid nitrogen. Then, 0.1 g of powder was lysed in 100 μL of SDS loading buffer (100 mM Tris-HCl pH 6.8, 4% SDS, 0.2% bromophenol blue, 20% glycerol, and 2% β-mercaptoethanol) at 95 °C for 8 min. Supernatants were separated on SDS-polyacrylamide gels. Anti-H3 antibody (A19645; ABclonal, Wuhan, China), anti-HA antibody (AE008; ABclonal, Wuhan, China), or anti-Actin antibody (A2319; ABclonal, Wuhan, China) were used. The images were captured using the GE Amersham Imager (AI680; GE, Pittsburgh, PA, USA).

### 4.4. RNA-Seq and Data Analysis

Twenty-six--day-old rosette leaves were collected and sent to Novogene for RNA extraction, cDNA library construction, and sequencing using Illumina NovaSeq 6000(Illumina, San Diego, CA, USA). RNA-seq data were analyzed as previously reported [58]. We used FastQC and MultiQC to check the read quality and aligned the clean reads with the *Arabidopsis* genome (TAIR10) using STAR. The EdgeR R package was used to analyze the DEGs between WT and *h3.3cr-4* (the parameters were set as follows: multiple-test corrected *p*-value < 0.05 and absolute log_2_ (fold change) >1.5). GO term enrichment of DEGs was analyzed using the R package clusterProfiler. The heatmap was drawn according to TPM values using the R package pheatmap (pheatmap: Pretty Heatmaps. R package version 1.0.12. https://CRAN.R-project.org/package=pheatmap, accessed on 11 October 2023).

### 4.5. I_2_-KI Staining

Mature pollen grains were collected from open flowers and stained with I_2_-KI solution (10 g KI and 5 g I_2_ were dissolved in 500 mL distilled water stored at 4 °C under dark conditions) according to the previous description [59]. Stained pollens were observed using the bright field of a Ti2-A inverted fluorescence microscope (Nikon, Tokyo Metropolis, Japan).

### 4.6. Alexander Staining

The pollen viability was observed by Alexander staining as described [60], with slight modifications. The anthers were fixed overnight in Carnoy’s fixative solution (anhydrous ethanol: glacial acetic acid, 3:1). The next day, anthers were stained in Alexander staining solution (100 mL solution configuration: 10 mL 95% ethanol, 25 mL glycerol, 1 mL 1% malachite green solution, 0.5 mL 1% orange G solution, 5 mL 1% acid fuchsin solution, 4 mL glacial acetic acid, and 54.5 mL distilled water) overnight without light. After being washed with water several times, stained anthers were observed with a Ti2-A inverted fluorescence microscope (Nikon, Tokyo Metropolis, Japan).

### 4.7. DAPI Staining

Mature pollen grains were collected from open flowers and stained with 1 μg/mL DAPI (MBD0015; Sigma, Shanghai, China) buffer. Stained pollens were observed using a TCS SP8 confocal laser microscope (Leica, Wetzlar, HE, Germany) for fluorescence observation. The fluorescence was excited using a 405 nm laser.

### 4.8. Differential Interference Contrast (DIC) Observation

The morphology of ovules or pistils was observed at stage FG7 after 36 h of emasculation. Flowers were fixed overnight in FAA solution (ratio of formalin: 70% ethanol: acetic acid of 2:1:1). Then, ovules or pistils were dissected and cleared using clearing solution (ratio of chloral hydrate: water: glycerol of 8:2:1). Cleared ovules or pistils were observed using a Ti2-A inverted fluorescence microscope (Nikon) with DIC optics.

### 4.9. Pollen Hydration

The stigmas were fixed on a clean glass slide. In this process, papillary cells of stigmas were prevented from being squeezed and deformed, which would affect subsequent observation. Then, the mature pollens were carefully placed on the papillary cells of stigmas by means of a glass capillary, and the morphological changes in the pollen within 0–20 min of pollination were observed under a Ti2-A inverted fluorescence microscope (Nikon).

### 4.10. Aniline Blue Staining

Aniline blue staining was carried out to explore pollen germination according to previous descriptions [60], with slight modifications. After 24 h of hand pollination, pistils were fixed overnight in Carnoy’s fixative solution. Fixed pistils were washed with PBS buffer three times for 20 min each time and treated with 5 M NaOH solution for 24 h. Then, the samples were rewashed with PBS buffer three times and stained with 0.1% aniline blue for 4 h under dark conditions. The stained pistils were observed using a TCS SP8 confocal laser microscope (Leica) for fluorescence observation. The fluorescence was excited using a 405 nm laser.

### 4.11. Scanning Electron Microscope (SEM) Observation

The SEM scanning was performed as previously described [61]. In brief, the stamen filaments were fixed on ice in the freshly prepared anterior fixative solution (2.5% glutaraldehyde, 0.1% Tween 20, and 100 mM PBS pH 7.4). The samples were vacuumed on ice, replaced with the fresh anterior fixative solution without Tween 20, and then fixed overnight at 4 °C. After being washed with PBS three times for 15 min each time, stamen filaments were dehydrated with 30%, 50%, 70%, 80%, 90%, and 100% gradient ethanol sequentially, for 15 min each. Then, they were dried in a carbon dioxide critical point dryer. After drying, the material was sprayed with gold and observed with a SEM (Hitachi-S3400N, Tokyo, Japan).

### 4.12. Semi-Quantitative and Real-Time Quantitative PCR

The hot phenol method was utilized to extract inflorescence RNAs [62]. The genomic DNAs were digested using DNase I (04716728001; Roche, Basel, Switzerland). Subsequently, cDNAs were synthesized using the Strand cDNA Synthesis Kit (R211; Vazyme, Nanjing, China). Semi-quantitative RT-qPCR was performed with 2 × Rapid Taq Master Mix (P222; Vazyme, Nanjing, China). Real-time quantitative PCR was performed with ChamQ SYBR^®^ qPCR Mix (Q311; Vazyme, Nanjing, China) on a real-time PCR instrument (Roche, Basel, Switzerland). The reference gene was *UBC* (AT5G25760). Three biological replicates were carried out. The primers are listed in Table S1.

## Data Availability

Sequencing data have been deposited in the China National Center for Bioinformation (CNCB) under accession number CRA013287. All the other raw data that support the findings of this study are available from the corresponding authors upon reasonable request.

## Supplementary Materials

The following supporting information can be downloaded at https://www.mdpi.com/article/10.3390/ijms25052549/s1.
