# Supplementary material for: The Histone Variant H3.3 Is Required for Plant Growth and Fertility in Arabidopsis
Source: Int J Mol Sci. 2024 Feb 22;25(5):2549. doi: 10.3390/ijms25052549 (PMC10932197; doi:10.3390/ijms25052549)
Supplement: Supplementary file 1 [file ijms-25-02549-s001.zip › Figure S1-S3.pdf]

International Journal of
Molecular Sciences

MDPI

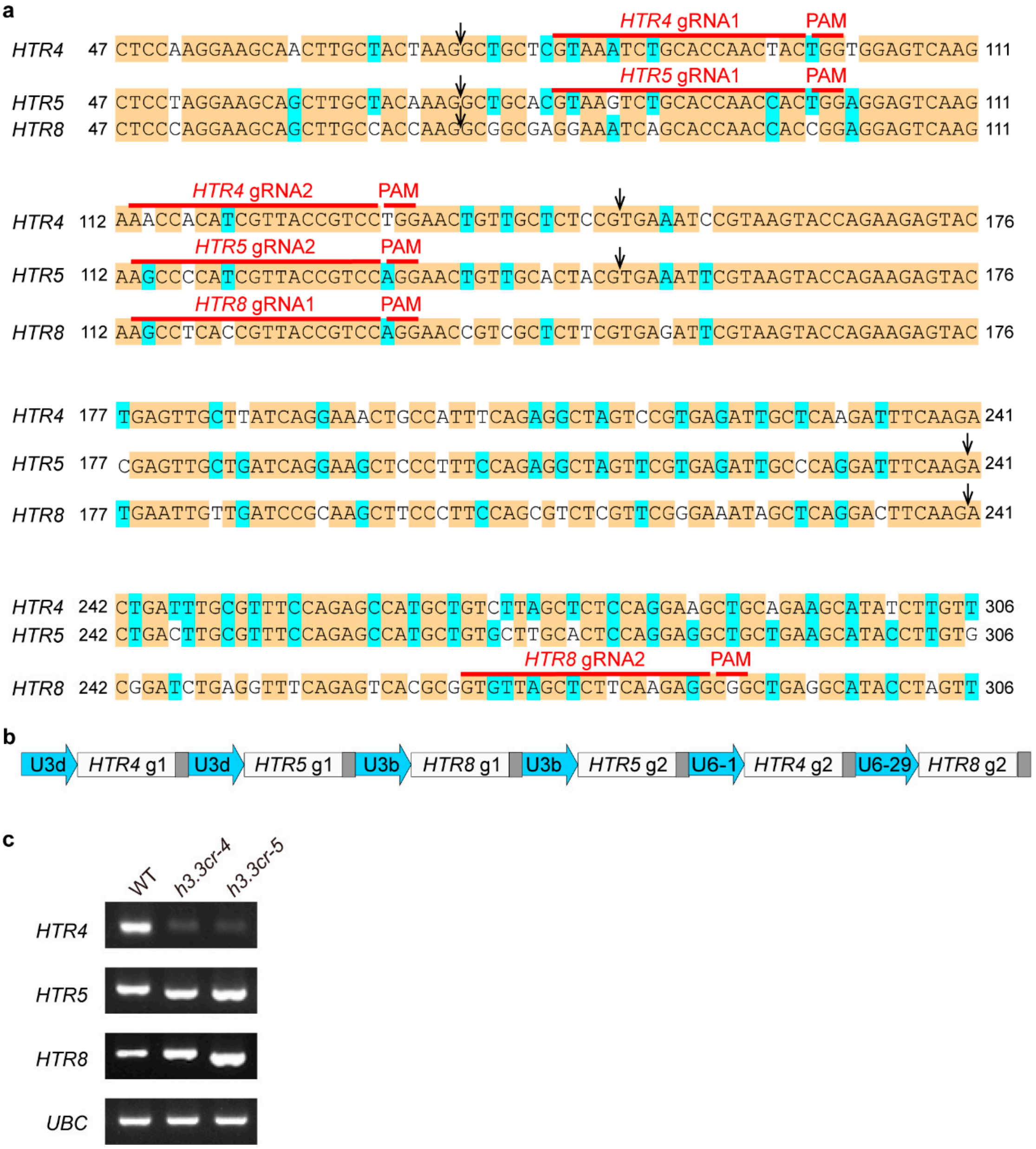

Figure S1. Schematic diagrams of *HTR4*, *HTR5*, and *HTR8* with the Cas9 targeting sites. (a) Design of gRNA target sites of *H3.3* CRISPR/Cas9 vector. The black arrows display the position of introns. The red line displays the gRNA sequence and PAM sequence. The earthy yellow background indicates completely consistent sequences and the blue background represents greater than or equal to 50% consistency. (b) Diagram of the six gRNAs constructed in the CRISPR/Cas9 vector. The blue arrows display the promoters. The white boxes display the gRNAs. The gray boxes display the gRNA scaffold and connected sequence. (c) Semi-quantitative RT-PCR analysis of full-length transcript (5′ to 3′ UTR) in WT, *h3.3cr-4*, and *h3.3cr-5*. *UBC* is used as an internal control. Primers are listed in Supplementary Table S1.

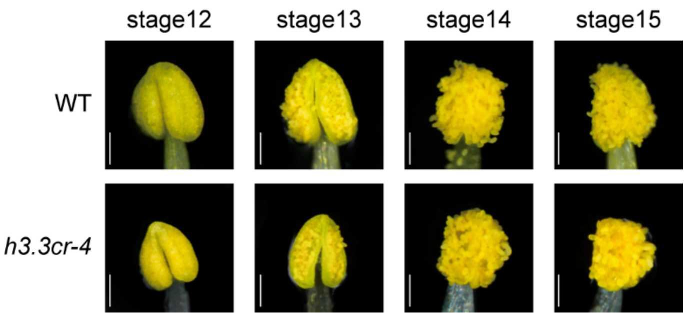

Figure S2. The anther dehiscence and pollen release in WT and *h3.3cr-4*. Anther and pollens of WT and *h3.3cr-4* at different flowering stages. Scale bar, 125 μm.

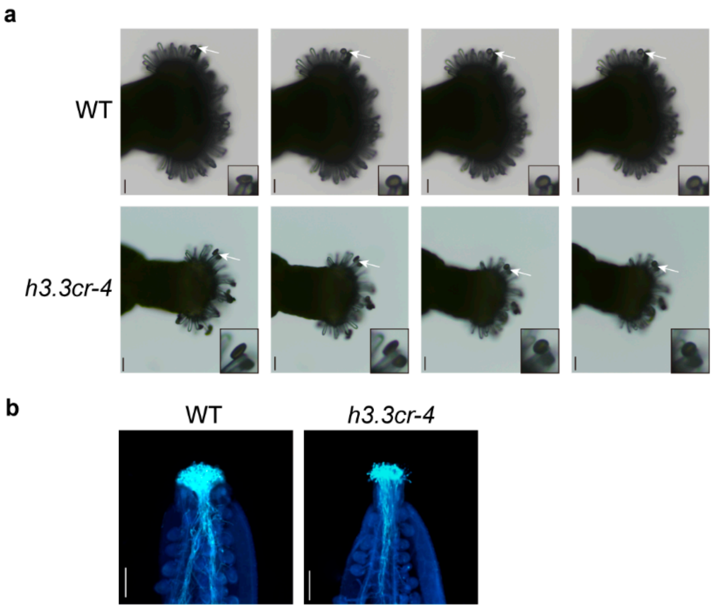

Figure S3. The hydration and germination of pollens in WT and *h3.3cr-4*. (a) Pollens hydration on the stigma. Scale bar, 100 μm. The insets (bottom right corner) exhibit the pollen where the white arrow points. (b) Pollen germination observed by aniline blue staining. Scale bar, 200 μm.
