# Supplementary figures and images for: The Histone Variant H3.3 Is Required for Plant Growth and Fertility in Arabidopsis
Source: Int J Mol Sci. 2024 Feb 22;25(5):2549. doi: 10.3390/ijms25052549 (PMC10932197; doi:10.3390/ijms25052549)

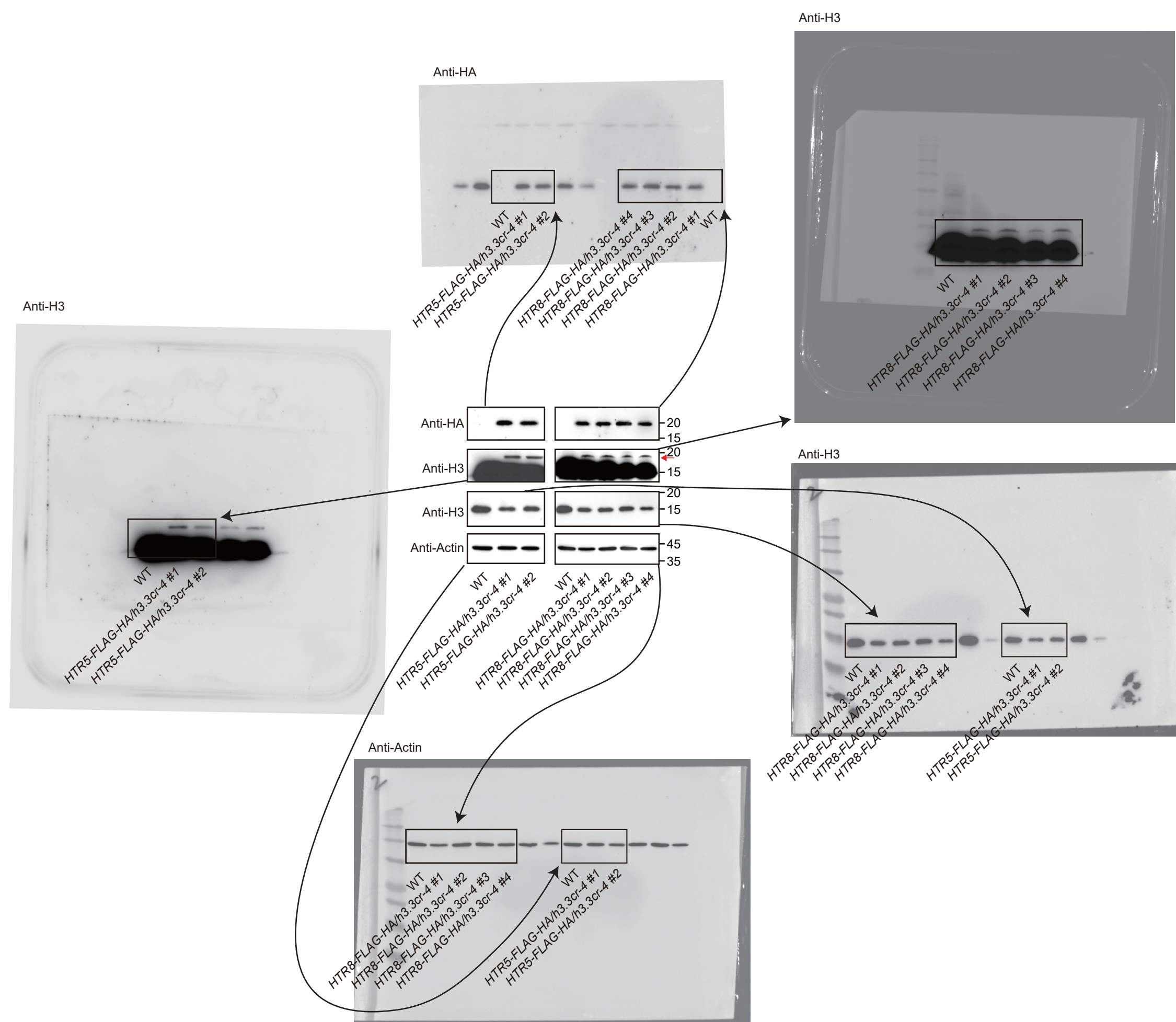

Supplement: Supplementary file 1 [file ijms-25-02549-s001.zip › ijms-2880224 Fig 2 original and uncropped WB images.pdf]

Fig S1 C

*UBC*

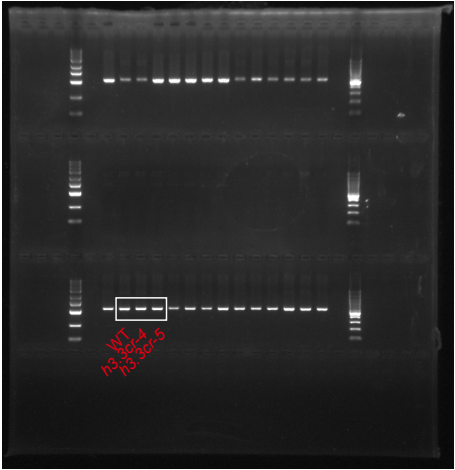

*HTR8*

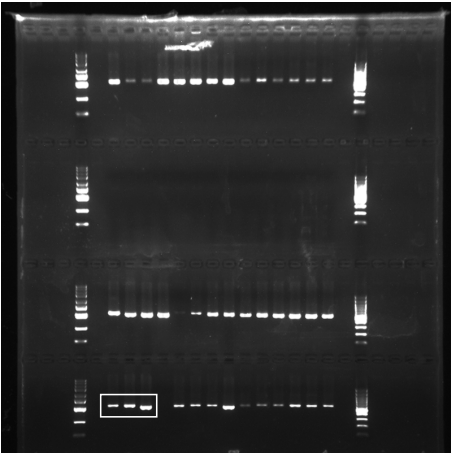

*HTR4*

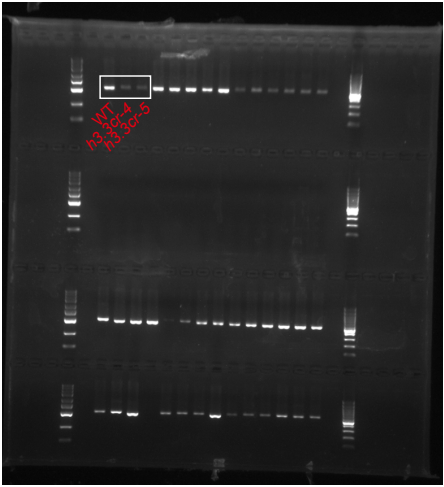

*HTR5*

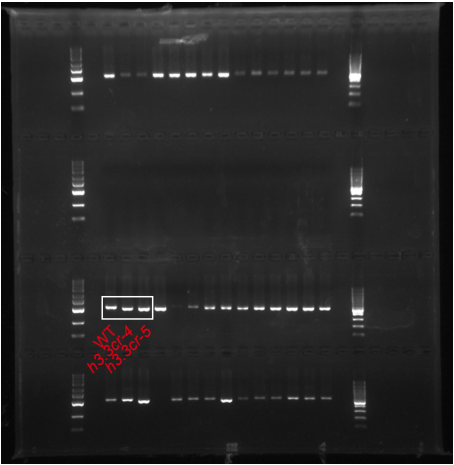

Supplement: Supplementary file 1 [file ijms-25-02549-s001.zip › ijms-2880224 Figure S1 source data.pdf]
